# Supplementary material for: A draft genome and transcriptome of common milkweed (Asclepias syriaca) as resources for evolutionary, ecological, and molecular studies in milkweeds and Apocynaceae
Source: PeerJ. 2019 Sep 20;7:e7649. doi: 10.7717/peerj.7649 (PMC6756140; doi:10.7717/peerj.7649)
Supplement: Supplemental Information 1 [file peerj-07-7649-s001.pdf]

SUPPLEMENTARY MATERIALS

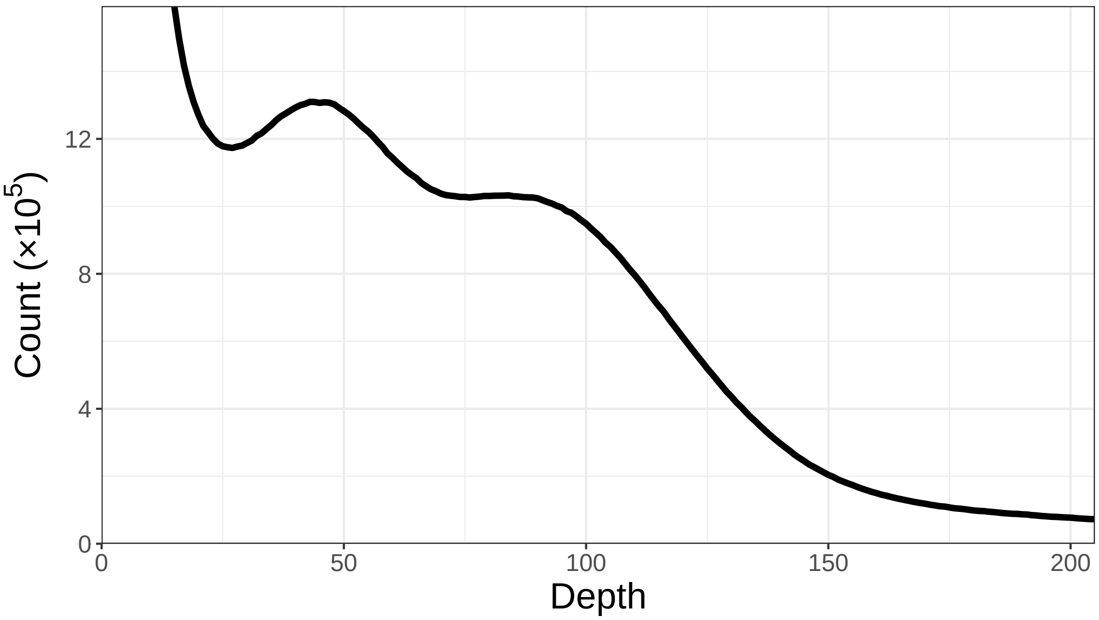

Figure S1: K-mer distribution of *Asclepias syriaca* genomic reads.

Depth is the number of times a certain 17 bp k-mer occurred in the genomic reads, and count is the number of different k-mers at that depth. K-mers with depths below 15 or above 205 are not shown. Within the read set analyzed, 629 million k-mers were unique. Peaks occur at 43× and 84× depth.

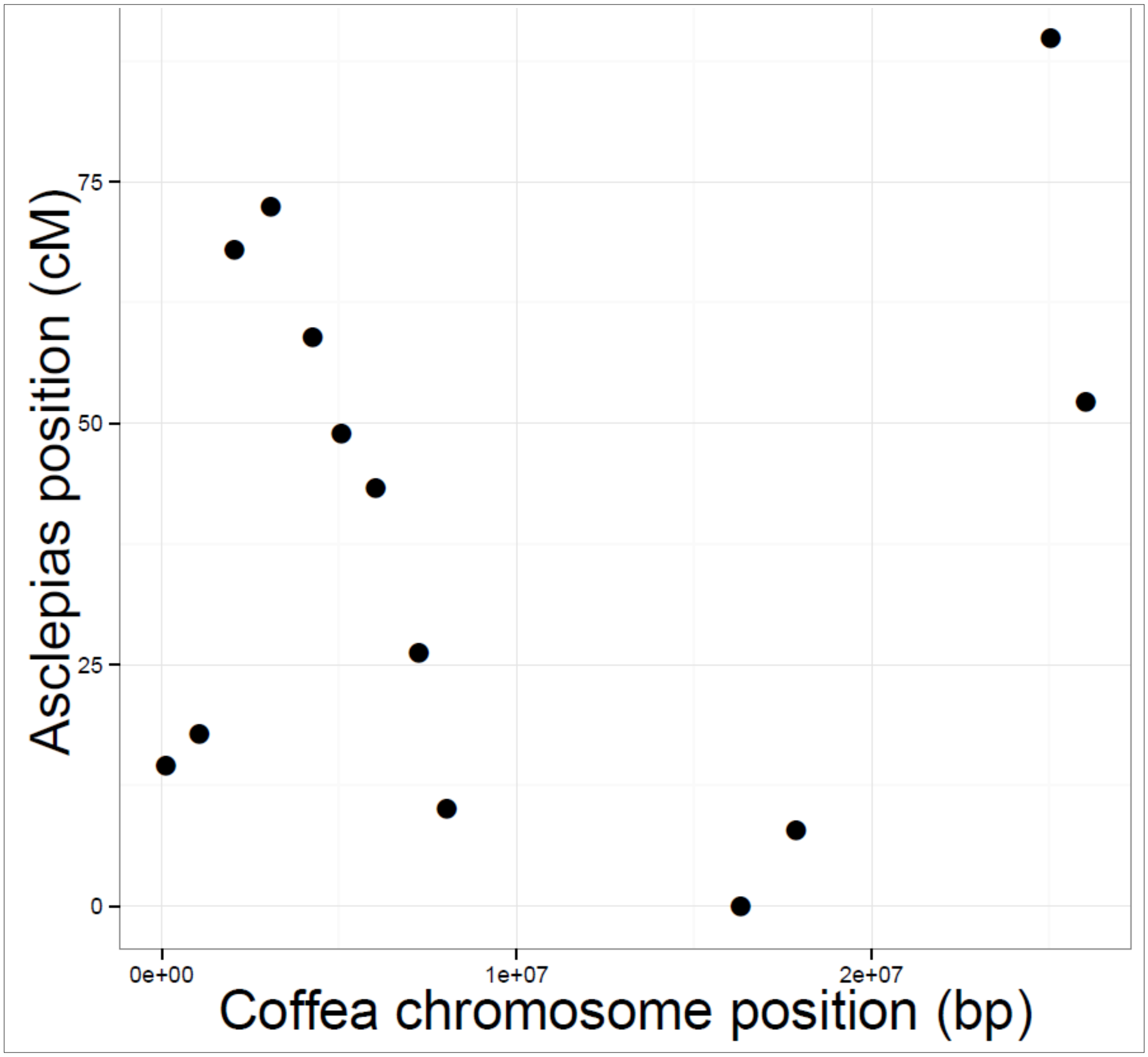

Figure S2: Synteny between *Asclepias* linkage group 2 and *Coffea* pseudochromosome 10.

A subset of scaffolds from *Asclepias* linkage group 2 mapped to their positions on *Coffea canephora* pseudochromosome 10, and ordered along the y-axis by recombination distance within *Asclepias*.

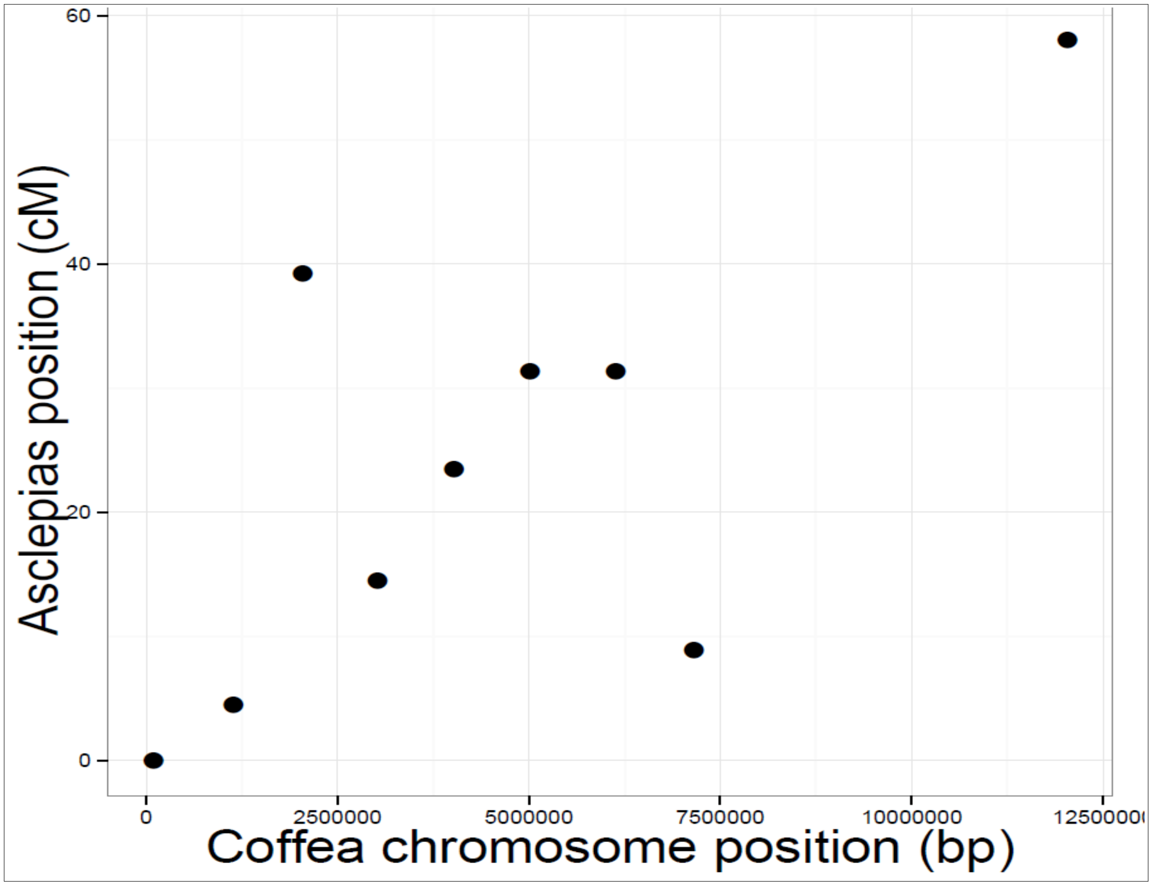

Figure S3: Synteny between *Asclepias* linkage group 8 and *Coffea* pseudochromosome 3.

A subset of scaffolds from *Asclepias* linkage group 8 mapped to their positions on *Coffea canephora* pseudochromosome 3, and ordered along the y-axis by recombination distance within *Asclepias*.

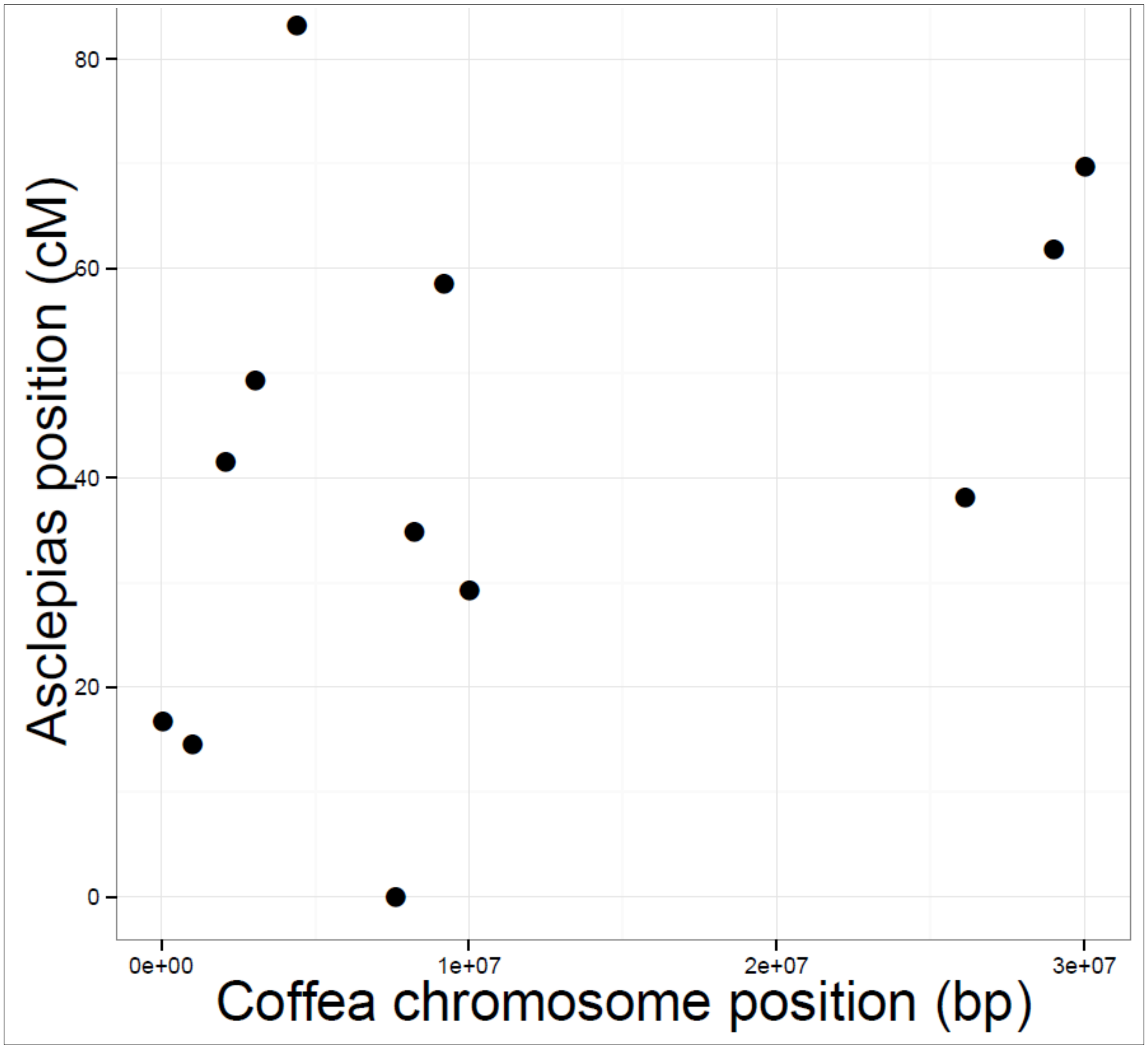

Figure S4: Synteny between *Asclepias* linkage group 4 and *Coffea* pseudochromosome 8.

A subset of scaffolds from *Asclepias* linkage group 4 mapped to their positions on *Coffea canephora* pseudochromosome 8, and ordered along the y-axis by recombination distance within *Asclepias*.

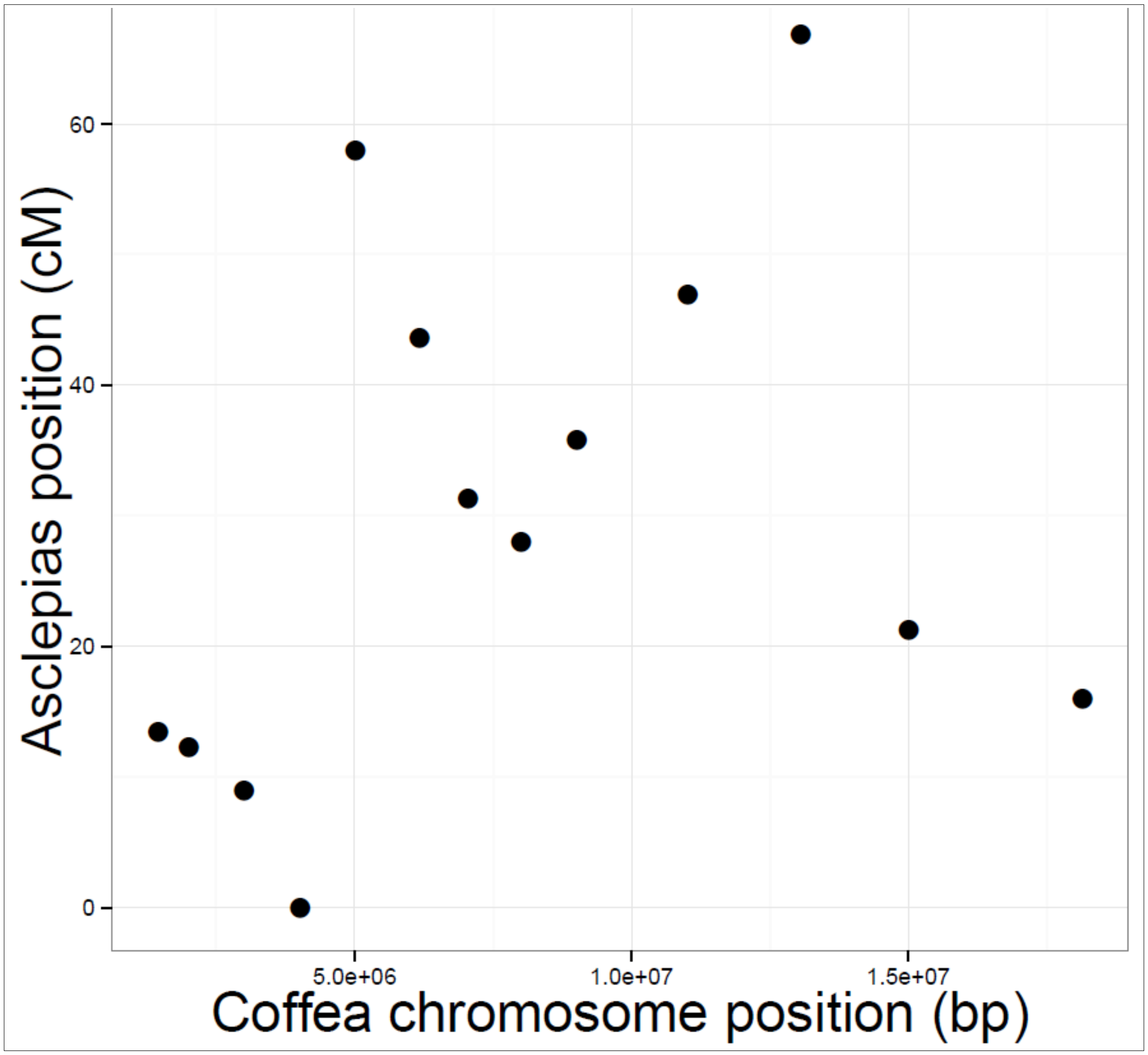

Figure S5: Synteny between *Asclepias* linkage group 6 and *Coffea* pseudochromosome 6.

A subset of scaffolds from *Asclepias* linkage group 6 mapped to their positions on *Coffea canephora* pseudochromosome 6, and ordered along the y-axis by recombination distance within *Asclepias*.

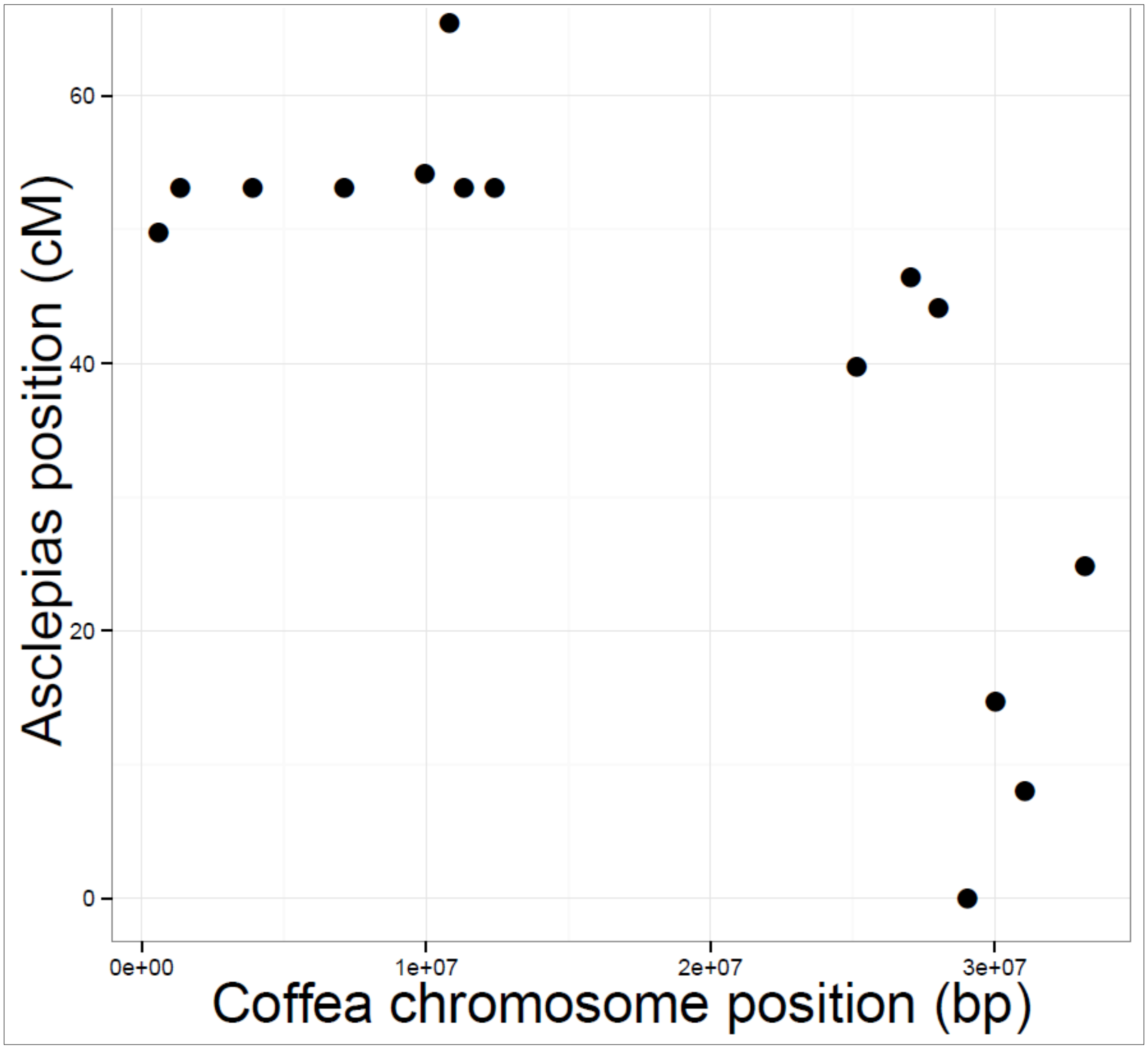

Figure S6: Synteny between *Asclepias* linkage group 7 and *Coffea* pseudochromosome 11.

A subset of scaffolds from *Asclepias* linkage group 7 mapped to their positions on *Coffea canephora* pseudochromosome 11, and ordered along the y-axis by recombination distance within *Asclepias*.

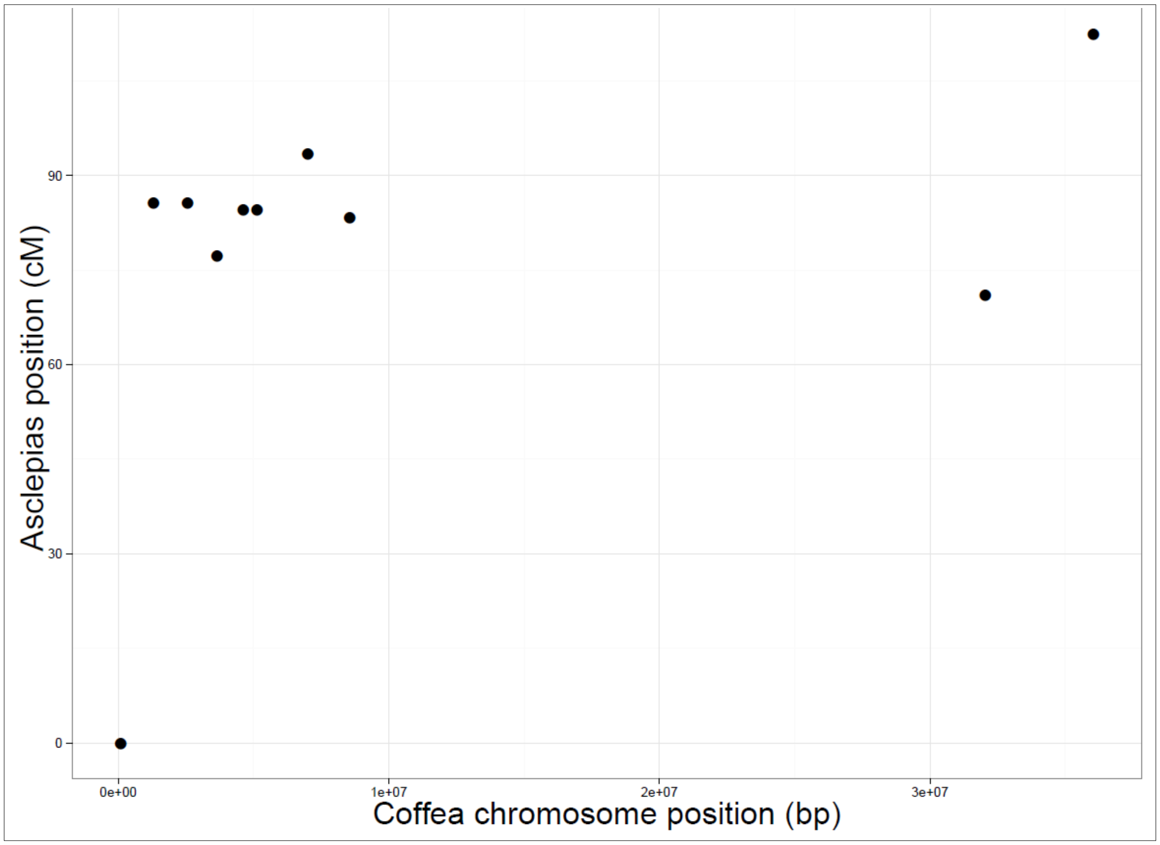

Figure S7: Synteny between *Asclepias* linkage group 9 and *Coffea* pseudochromosome 1.

A subset of scaffolds from *Asclepias* linkage group 9 mapped to their positions on *Coffea canephora* pseudochromosome 1, and ordered along the y-axis by recombination distance within *Asclepias*.

Table S1: Shared orthogroups among *Asclepias*, *Catharanthus*, *Coffea*, and *Vitis*. Values along the diagonal are the number of orthogroups found within that genus.

	Asclepias	***Catharanthus***	***Coffea***	***Vitis***
Asclepias	9,837	9,275	9,439	8,753
Catharanthus		12,709	12,111	11,072
Coffea			13,233	11,688
Vitis				12,117

Table S2: Genomic coordinates of *Asclepias* P5βR genes.

Under **Scaffold ID** the linkage group of the scaffold is preceded by "LG." These scaffold IDs refer to sequences provided in the Oregon State University institutional archive (Weitemier, 2017; doi.org/10.7267/N9M61HDR). **NCBI ID:** The scaffold IDs as given in NCBI/GenBank. **Called:** Whether the gene prediction consensus accurately predicted the correct exons. The prediction that failed did predict a gene product, but included exons from adjacent genes. Ψ-progesterone 5β-reductase was accurately predicted to not produce a product.

Gene	Scaffold ID	NCBI ID	Start	Stop	Called
Progesterone 5β-reductase 1	LG11_scaffold_m502	MSXX01042206.1	31537	33459	No
Ψ-progesterone 5β-reductase	LG11_scaffold_m502	MSXX01042206.1	33942	34876	NA
Progesterone 5β-reductase 6	LG00_scaffold217668	MSXX01007058.1	1305	136	Yes
